# 121 Acute Stress Disorder and Post-traumatic Stress Disorder in the Outpatient Burn Population

**DOI:** 10.1093/jbcr/irac012.123

**Published:** 2022-03-23

**Authors:** Kiran U Dyamenahalli, Heather Carmichael, Arek J Wiktor

**Affiliations:** University of Colorado, Denver, Colorado; University of Colorado, Denver, Colorado; University of Colorado Burn Center, Aurora, Colorado

## Abstract

**Introduction:**

Early screening and intervention for acute stress disorder (ASD), diagnosed within 30 days of the inciting trauma, and post-traumatic stress disorder (PTSD), diagnosed after 30 days, are quality metrics in burn care. However, a considerable knowledge gap remains surrounding these psychological conditions in the *outpatient* burn setting. In this study, we assessed the effectiveness of ASD and PTSD screening at an academic burn center and identified risk factors for their development.

**Methods:**

A retrospective cohort study of all patients treated at our ABA-verified burn center’s outpatient clinic, between July 2016 and August 2019, was undertaken. Adult patients with flame, flash, contact, or scald burns who were initially evaluated in the outpatient setting were included. ASD and PTSD were assessed using validated screening tools (ASDS and PCL-5, respectively). ASD/PTSD screening rate, screening tool appropriateness, and subsequent interventions were tracked, along with age, gender, % total body surface area (TBSA) burned, burn mechanism, operative intervention, psychiatric history, substance abuse history, and co-morbidities. Chi-square and Mann-Whitney *U* tests were used for univariate analysis of categorical and continuous variables, respectively.

**Results:**

The analysis included 2494 clinic encounters and 1147 unique patients. Patients were screened for ASD or PTSD at 94.8% of encounters. Median age was 36 years (range of 18 to 94 years), 57.6% of patients were male (n=661), and median TBSA burned was 1% (range of 0.1 to 12%). Among all screens, the appropriate screening tool was applied 88.5% of the time. For all encounters, positive screening rates for ASD and PTSD were 13.2% (n=286) and 14.6% (n=48), respectively. Risk factors for positive ASD screens included a history of substance abuse (OR 1.9, *p*=0.03) and history of psychiatric illness (OR 2.6, *p*=0.002). Similarly, risk factors for positive PTSD screens included a prior positive ASD screen (OR 9.5, *p*=0.001), a history of substance abuse (OR 2.1, *p*=0.04), and a history of psychiatric illness (OR 3.3, *p*=0.002). Age, gender, burn mechanism, TBSA burned, and need for operative intervention did not predict positive screens. The intervention rate for positive PTSD screens by referral, counseling, or medication, was only 7.9%.

**Conclusions:**

Demographics and burn severity do not appear to predict development of ASD or PTSD in the outpatient burn population. In contrast, a history of substance abuse or psychiatric illness warrant further attention. Despite consistent use of validated screening tools, these conditions remain under-treated in the outpatient setting, indicating a need for resource-expansion.

